# How to encourage patients to come for the eye services they need

**Published:** 2012

**Authors:** Boateng Wiafe

**Affiliations:** Regional Director for Africa, Operation Eyesight Universal

**Figure:**
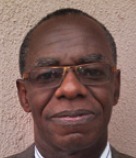
Boateng Wiafe

Encouraging people to seek out eye care services involves raising awareness about eye care in general, raising awareness about the existence and benefits of a particular eye clinic or hospital, and being clear about the costs involved. People in the community may be unaware of the existence of eye care services; they may also think that they do not need the services, or that they will not be able to afford the treatments. People may fear eye surgery; they may also believe that some eye disorders cannot be prevented or cured.

There are many established ways of addressing misconceptions and lack of information in the community. Increasing people's awareness about eye care is necessary, but not enough: we also want them to come to our eye centre, hospital, or clinic to get the eye care they need.

If we want to successfully change people's behaviour, we must understand who we are trying to reach (see page 22):

- What do they already know?
- What opinions do they hold about eye care and related issues, such as eye care for older people or women?
- What motivates them? What is important to them?
- Who has influence with this community?

This information can help us to design a programme of information, education, and communication (IEC) activities aimed at convincing people to change their own health behaviour and make use of the health services we offer. When these activities take the form of advertising campaigns, this is known as ‘social marketing’.

**Figure:**
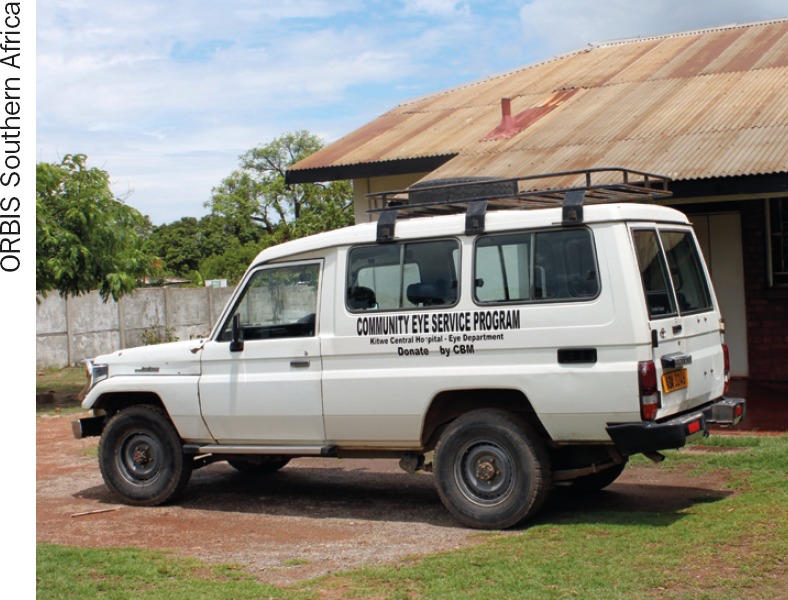
Community outreach will raise awareness about your eye programme. ZAMBIA

**Figure:**
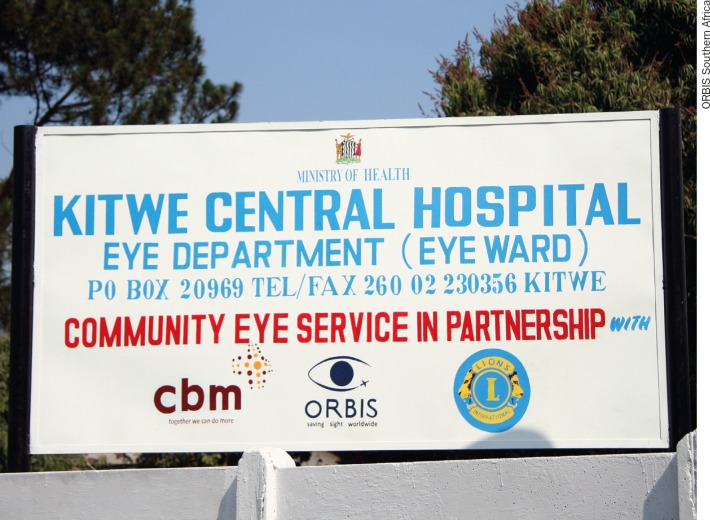
This sign, near the main entrance, ensures that everyone visiting the main hospital knows that eye care is also available here. ZAMBIA

Being successful at IEC or social marketing does not always require a lot of money. It does, however, require careful thought and planning. Here are a few ideas and suggestions for all your communication activities.

**‘Patients who have received good eye care can convince many others to come for treatment’**

1. **Posters and promotional materials.** If you want to create posters, make sure they are appropriate for the community you want to reach and that the language and pictures communicate the right messages. Think: who are you trying to reach? What motivates them? Who do they respect?
  It is always advisable to test the posters first by showing them to people from the community you are trying to reach. Ask them what they think the posters mean, and try to determine whether they feel more motivated to change their behaviour after seeing the posters. Think about where people are most likely to see the posters – for example at bus stops, near shops, or in community centres.
  Ask for help from others who have done similar work, for example people working on HIV/AIDS or TB campaigns.
2. **Interpersonal communication.** You could teach community leaders or other respected community members about eye care, where to go for help, and how much this will cost. They can share the new information with others. Patients who have received good eye care are also very important as they can convince many others to come for treatment. Consider inviting them to talk about their experiences at community gatherings.
3. **Newspapers, magazines, radio, and television.** Journalists are often looking for new ideas or community initiatives to support, and will be happy to tell their readers, listeners, or viewers about the importance of eye care. For example, you can tell them that many older people in the community are going blind from cataract, and that a simple and affordable operation could restore their sight. Radio serials (soap operas) have been used successfully in many part of the world to communicate health care information to listeners. The writers and producers of these series are often looking for ideas for stories and may be able to include eye care messages. A story about what happened when someone could see again for the first time after a cataract operation might make an exciting and dramatic episode!
4. **Community outreach programmes.** Outreach programmes such as eye screening and community-based rehabilitation can be very helpful. You will raise awareness about your eye programme simply by being present in the community, and you will also have an opportunity to meet and work with local organisations such as religious groups, local charities or non-governmental organisations. These groups often have good access to community members who may be willing to help with your social marketing campaigns. They can also help to provide infrastructure and other logistical support for your outreach activities.
5. **Targeting key groups.** Promote eye care in general and your services in particular to key groups. For example, if you wanted to encourage older adults to come to you for cataract surgery, you could try to reach pensioner groups, old age homes, and so on (see panel, right). You could visit them to tell them about your services. If possible, invite former patients to talk about their positive experiences.

Make contact with other health care workers; they may help you educate community members and assist with case finding. For example, you could give cataract information leaflets to physicians or nurses who work with older people.

## Other barriers

Listen to what the community says about the obstacles they face coming to the clinic: transport, safetly, cost, etc. Do what you can to help and explain where they can go for additional support.

Cost is an important issue. Many people are afraid that they will not be able to afford eye care services. Include all the costs clearly in any promotional materials.

Include fee waivers, for example for low-income groups. Specify where your services are covered by national or local health insurance schemes, or any private schemes that are administered through your hospital or provider.

At the clinic itself, make sure patients can see what the different costs are, such as the registration fee, outpatient fees, and cataract surgery fees.

**Getting patients into the eye care system: as easy as ABCD!**

**A Awareness creation.** Increase the community's understanding of eye care and when they should seek help. Ensure they know about your eye service and how to access it. There should be a clear referral pathway.

**B Best service.** Offer quality services. Encourage staff to do their best. Patients who have had positive experiences will encourage others to come.

**C Cost.** Ensure that patients understand all the costs involved, also the indirect costs such as travel expenses.

**D Distance.** Make sure the services are as close to the community as possible. Be in the community!

**FROM THE FIELD: Reaching out to older people in the community**

**Figure:**
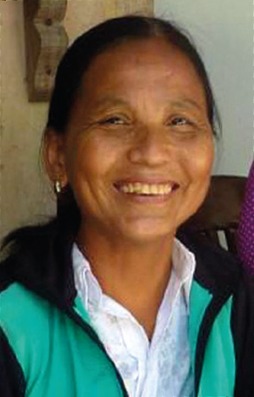
**Nguyen Thi Hien** lives and works in the Que Phong community, which is located in Quang Nam province in Viet Nam. She has provided care to the members of her local community for more than thirty years, ten of those years as a Village Health Worker. Although Ms Hein was a passionate and skilled volunteer she had never received any training in Primary Eye Care.

In 2006, Ms Hien was invited to take part in a primary eye care training course. Alongside other village health workers, she learnt about blindness prevention and how to provide basic eye care services to the local community, including diagnosis and treatment of simple eye problems and how to refer patients for cataract and other eye operations.

After the course, Ms Hien felt inspired to raise awareness about blindness prevention. She began working with existing ‘elder clubs’ in her community to educate the elderly members of the community about cataract surgery. The first time she visited the clubs, the members knew very little about cataract and blindness prevention. To begin with, they were doubtful about what Ms Hein was saying. After much persistence, several patients agreed to travel with her to the district hospital for cataract surgery.

The operations were a big success and word quickly spread through the elder clubs of the availability and quality of the cataract surgery. More patients became interested in surgery and Ms Hein provided them with advice and support. Ms Hein invited the growing number of patients who had received cataract surgery to talk to the elder clubs, sharing their experience with other members and explaining how cataract surgery had changed their lives.

Ms Hien is now the head of Gia Cat Tay village, and continues to raise awareness about eye care services and to refer many patients to the district hospital for surgery.

**Figure:**
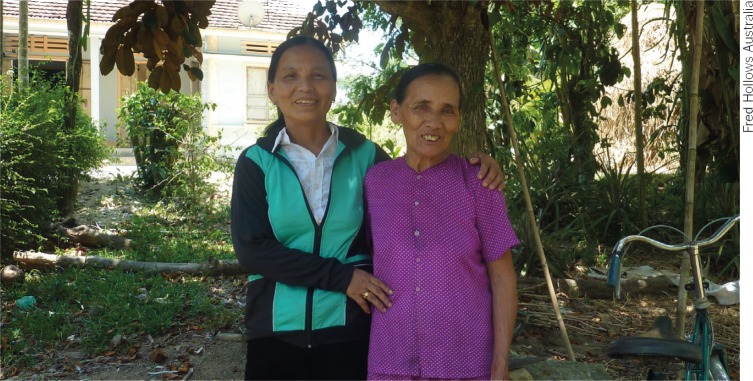
Ms Hien (left) with one of the older women she has supporteded to undergo cataract surgery. VIET NAM

